# *Amphilagus plicadentis* (Lagomorpha, Mammalia) from the Tagay locality (Olkhon Island, Baikal region, Eastern Siberia)

**DOI:** 10.1007/s12549-022-00554-y

**Published:** 2022-11-28

**Authors:** Margarita A. Erbajeva, Gudrun Daxner-Höck, Thomas Mörs

**Affiliations:** 1 Dobretsov Geological Institute, Siberian Branch, Russian Academy of Sciences, Sahianova Str. 6a, Ulan-Ude, 670047 Russia; 2 Seekirchen, Austria; 3 Natural History Museum Vienna, Burgring 7, 1010 Vienna, Austria; 4 Department of Palaeobiology, Swedish Museum of Natural History, P.O. Box 50007, 10405 Stockholm, SE Sweden; 5 Bolin Centre for Climate Research, Stockholm University, Stockholm, Sweden

**Keywords:** Lagomorpha, *Amphilagus*, Miocene, Olkhon Island, Baikal region, Eastern Siberia

## Abstract

New excavations in the Tagay locality have revealed the presence of *Amphilagus plicadentis* Erbajeva, 2013, which belongs to the palaeolagine Lagomorpha rather than leporids, previously referred to *Procaprolagus* sp. (Logachev et al., 1964). Herein, we report a comprehensive morphological description of this species and compare it with the other known species of the genus *Amphilagus*. The Tagay lagomorph is conspecific with the Early Miocene species *Amphilagus plicadentis* found at locality Unkheltseg (UNCH-A) (biozone D; Early Miocene) in the Valley of Lakes, Central Mongolia. The Tagay specimens suggest a more advanced evolutionary stage of *A. plicadentis* and an age around Early/Middle Miocene transition.

**Supplementary Information:**

The online version contains supplementary material available at 10.1007/s12549-022-00554-y.

## Introduction

Fairly rich mammalian associations at the Tagay Bay of Lake Baikal were first discovered by Kitaynik (1958) and later reported by Logachev et al. (1964), including additional material. The lagomorphs were attributed to the leporid species *Procaprolagus* sp. Considering the presence of *Cricetodon* cf. *sansaniensis* and *Monosaulax* sp., I.M. Gromov (in Logachev et al. 1964) identified the Tagay fauna as Middle to Late Miocene. Other researchers confirmed this, in particular Trofimov, Khozatski and Yakovleva (in Logachev et al. 1964). All taxa of small mammals from the Tagay Bay, initially reported by Logachev (1964), were later re-studied by Pokatilov (1994). Pokatilov (1994) also erroneously included the Oligocene lagomorph *Desmatolagus* sp., which never crossed the Oligocene-Miocene boundary, to the faunal list.

In 2014, new material was collected by an international field party and it turned out that the lagomorph remains from Tagay belong to the palaeolagine genus *Amphilagus*, rather than to leporids. In the Baikal region an another lagomorph is known, it is *Amphilagus tomidai* Erbajeva, Angelone, Alexeeva, 2016 found in the Aya Cave site which contained Middle Miocene-Plio- Pleistocene deposits. The representatives of the genus *Amphilagus* were discovered in different regions of Asia, e.g. Russian Siberia, Japan, China, Kazakhstan and Mongolia (Tomida and Goda 1993; Erbajeva 1994; Qiu 1996; Erbajeva and Filippov 1997; Wu et al. 1998; Filippov et al. 2000; Erbajeva and Daxner-Höck 2001, 2014; Erbajeva 2013). The genus *Amphilagus* Pomel, 1853 appeared in Europe during the late Oligocene. It includes the species *Amphilagus antiquus* Pomel, 1853 (Viret 1929; Fostowicz-Frelik 2016), *Amphilagus ulmensis* Tobien, 1974 and *Amphilagus wuttkei* Mörs and Kalthoff, 2010. During the Oligocene and Miocene, the genus diversified and widely dispersed in Western, Central and Eastern Europe (Major 1899; Viret 1929; Engesser 1972; Topachevsky 1987; Angelone 2009a, 2009b; Harzhauser et al. 2011; Fostowicz-Frelik et al. 2012, 2016). Later amphilagines dispersed in and occupied vast territories of Asia, e.g. Kazakhstan (Zaisan basin), the south of East Siberia, Central Mongolia (Valley of Lakes) and proceeded to China and Japan (Tomida and Goda 1993; Wu et al. 1998). Amphilagines were recently discovered in Miocene faunas of Mongolia as well. Being rather abundant and diverse, they represent there the three taxa *Amphilagus orientalis* Erbajeva, 2013, *Amphilagus plicadentis* Erbajeva, 2013 and *Amphilagus magnus* Erbajeva, 2013. One of them, namely *Amphilagus plicadentis,* is found in the Tagay fauna of East Siberia.

## Material and methods

The lagomorph fossils were collected by screen washing of sediments from three fossiliferous horizons (7, 9, 10) of the Tagay section; locality and rodent fauna are discussed in Daxner-Höck et al. (2022a, 2022b, 2022c, 2022d, this issue) and Mörs et al. (2022, this issue). The lagomorph collections are stored at the Dobretsov Geological Institute, Siberian Branch, Russian Academy of Sciences (GIN SB RAS), Ulan-Ude, Russia.

The dental terminology used here follows Tobien (1974) and Lopez Martinez (1989). The measurements, made by standard methodology, are presented in Table 1 in mm. The classification of Lagomorpha follows Gureev (1964), accordingly the genus *Amphilagus* belongs to subfamily Amphilaginae Gureev, 1953 of the family Palaeolagidae Dice, 1929.

**Table 1:** Measurements (in mm) of *Amphilagus plicadentis* teeth from the Tagay-1 section (Olkhon Island, Baikal region, Siberia)

Specimens	n	M	min	max
P3 L	1	1.85		
P3 W	1	3.5		
P4 L	5	1.87	1.75	2.1
P4 W	3	3.6	2.75	4.2
M1 L	1	1.75		
M1 W	1	3.85		
M2 L	1	1.7		
M2 W	1	3.5		
p3 L	1	1.7		
p3 W	1	2.2		
p4 L	1	2.35		
p4 W	2	2.25	2.25	2.25
m1 L	4	2.08	2.0	2.25
m1 W	3	2.23	2.2	2.25
m2 L	1	2.25		
m2 W	1	2.5		

## Abbreviations

GIN SB RAS: collections of Dobretsov Geological Institute, Siberian Branch, Russian Academy of Sciences, Ulan-Ude, Russia
P, M: premolars and molars of the upper dentition
p, m: premolars and molars of the lower dentition
I^1^, I^2^: upper first and second incisors
n: number of specimens
min, M, max: minimum, mean and maximum observed ranges
L, W: length, width
l, r: left, right
MN: European Neogene Mammal zone (Steininger, 1999)

## Systematic Palaeontology

Class Mammalia Linnaeus, 1758

Order Lagomorpha Brandt, 1855

Family Palaeolagidae Dice, 1929

Subfamily Amphilaginae Gureev, 1953

Genus *Amphilagus* Pomel, 1853

**Fig. 1:**
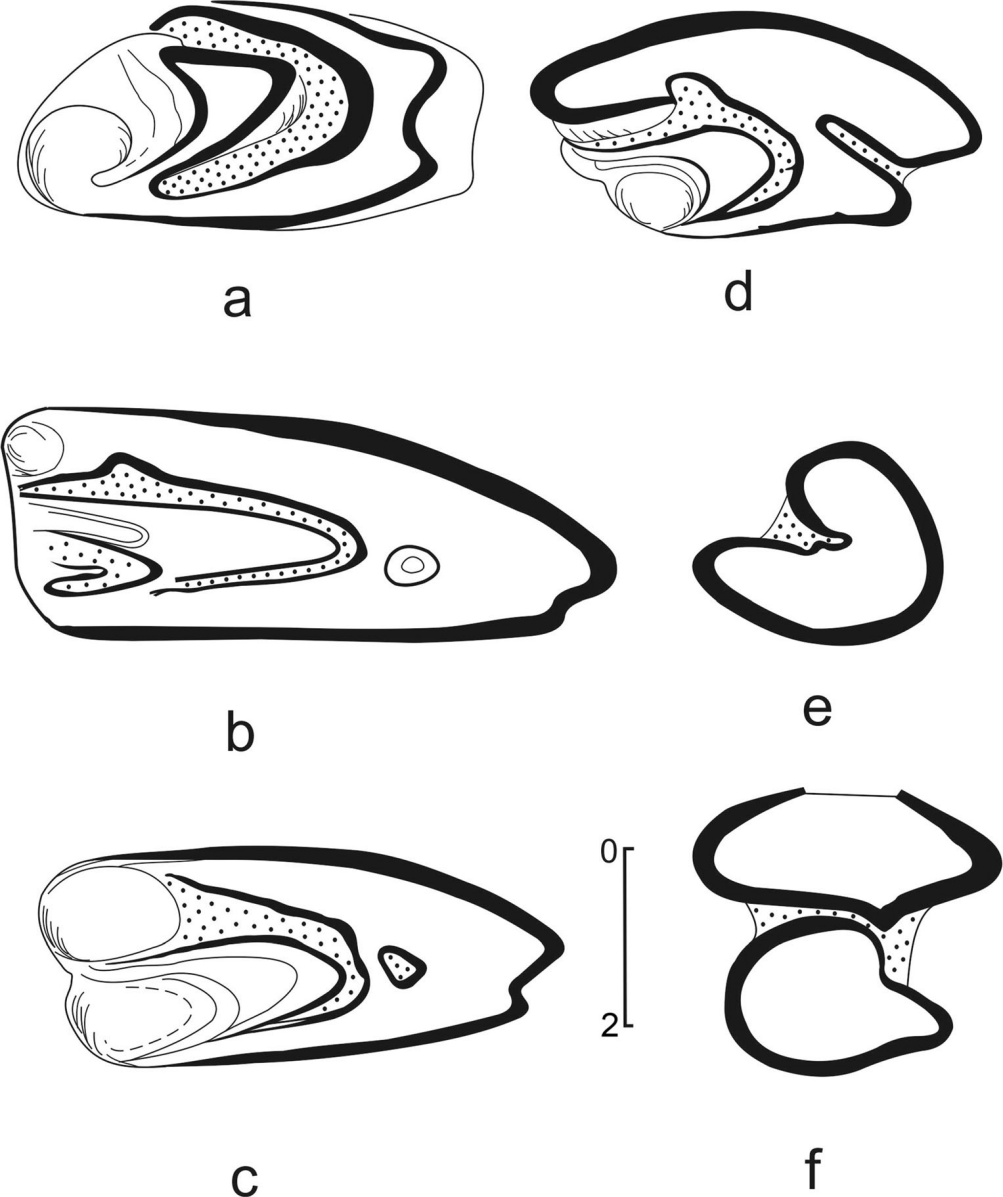
*Amphilagus plicadentis* Erbajeva, 2013 from the Tagay site of Olkhon island, Baikalian region. **a** Right P3 (GIN Nr 2014/0003/1), horizon 9. **b** Left P4 (reverse) (GIN Nr 2014/0003/5), horizon 10. **c** Right M1 (GIN Nr 2014/0003/7), horizon 7. **d** Right M2 (GIN Nr 2014/0003/8), horizon 9. **e** Left p3 (GIN Nr 2014/0003/9), horizon 9. **f** Right p4 (GIN Nr 2014/0003/10), horizon 9.

*Amphilagus plicadentis* Erbajeva, 2013

(Fig. 1a-f, Tables 1 and 2)

**Table 2:** *Amphilagus* upper and lower teeth measurements (in mm)

Nr	Specimens	*Amphilagus plicadentis* (Tagay)		*Amphilagus plicadentis* (Mongolia)		*Amphilagus tomidai* (Aya Cave)		*Amphilagus magnus* (Mongolia)		*Amphilagus orientalis* (Mongolia)		*Amphilagus wuttkei*		*Amphilagus antiquus*		*Amphilagus antiquus* (Herrlingen, Germany)	
												After Mörs and Kalthoff 2014)					
		n	M	n	M	n	M	n	M	n	M	n	M	n	M	n	M
1	P3	1	1.85	1	2.5	2	1.95	1	2.3	6	2.2	1	1.96	18	1.8		
2	P4	5	1.87			6	2.51	3	2.26	2	2.15	1	2.0	28	1.85	2	2.03
3	M1	1	1.75			5	2.1	3	2.1	2	2.2	1	1.84	20	1.6	1	1.66
4	M2	1	1.7			2	2.0	1	2.0	3	1.86	1	1.57	23	1.45		
5	p3	1	1.7	1	1.75	1	1.7	1	1.5	5	1.3						
6	p4	1	2.35			4	2.95	7	2.53	3	2.53					3	2.22
7	m1	4	2.23			8	2.6	8	2.4	2	2.35					1	2.1
8	m2	1	2.25			5	2.85	3	2.55	3	2.6						

2013 *Amphilagus plicadentis* Erbajeva, M. A., p. 315.

2014 *Amphilagus plicadentis* Erbajeva, M. A. and Daxner-Höck, G., p. 231.

**Locality:** Tagay, layers 7, 9 and 10 of the Tagay section Olkhon Island, Baikal region, south Eastern Siberia,

**Stratigraphic range**: Tagay Formation; Early/Middle Miocene transition.

**Material**: 1 P3 (GIN Nr 2014/0003/1), 5 P4 (GIN Nr 2014/0003/2-6), 1 M1 (GIN Nr 2014/0003/7), 1 M2 (GIN Nr 2014/0003/8), 1 p3 (GIN Nr 2014/0003/9), 2 p4 (GIN Nr 2014/0003/10-11), 3 m1 (GIN Nr 2014/0003/12-14), 1 m2 (GIN Nr 2014/0003/15), 1 m1 trigonid (GIN Nr 2014/0003/16), 1 I (GIN Nr 2014/0003/17), 1 I^2^ (GIN Nr 2014/0003/18), 1 R I_1_ (GIN Nr 2014/0003/19), 1 L I_1_ (GIN Nr 2014/0003/20).

Measurements are given in Tables 1 and 2.

### Description

The newly described material mainly consists of isolated teeth, few of them are damaged. Most material is derived from horizon 9 (14 specimens), three specimens from horizon 7 (P4r, P4l, M1r) and three from horizon 10 (P4r, I^1^, I^2^) (see Supplementary Material 1). No remains of P2 were found. As in all amphilagines, the upper teeth (P3-M2) have three roots - one large internal and two small lateral roots. The lower dentition (p4-m2) has two roots, except of for p3. Most lower teeth represent adult individuals.

**P3** (Fig. 1a): The occlusal surface is typical for all known amphilagine taxa in having three cones, separated by anterior reentrants, a deep internal one (paraflexus) filled with abundant cement and a smaller external reentrant (mesoflexus) without cement. The internal cone (protocone) is largest with long anteroloph stretched to the middle of the tooth width. The middle cone (paracone) is relatively large, having an anterior bulbous shaped triangle and a posterior narrow “isthmus“, connecting it with the small external cone. The latter is small-sized and has a round shape, separated from the middle cone by a rather shallow mesoflexus without cement. The labial part of P3 shows a smooth rounded margin, the lingual side of the tooth has a shallow and broad inflection (hypoflexus) without cement. The hypocone with a sharp edge is small-sized. The enamel band of the tooth is well developed across whole tooth margins.

**P4** (Fig. 1b): The structure of P4-M2 is typical for amphilagines. On the occlusal surface the crescentic valley is well developed, and it is filled with cement. The specimens of young and mid-age individuals display quadrangular shape with shallow hypostria, which extend almost to the crown base. Probably with aging, the hypostria deepens and enters the occlusal surface and in worn teeth of adult specimens it remains as a small enamel islet. The P4 of adult specimens is much longer on the labial side than on the lingual one, which becomes gradually narrow and V-shaped. With age this tooth widens, having a prominent labial part, and a concave lingual one; parafossete and metafossete are long and filled with cement.

**M1** (Fig. 1c): The molar is slightly smaller than P4; in adult specimens, the paracone and metacone are equally-sized; as in P4 the labial part of the tooth is longer than the lingual one. The parafossete is filled with cement and separated from the enamel islet. The enamel band of the tooth is thick on the anterior and posterior margins, mostly on the lingual part but in the internal part of the tooth the enamel band is much thinner.

**M2** (Fig. 1d): The smallest tooth among molariforms, of reverse trapezoidal shape has smooth rounded borders. It is slightly concave in the middle part of the occlusal surface. The enamel band is thick on the anterior and slightly less on the posterior margin. Hypostria is deep, entering almost 1/3 of the tooth width, filled with cement.

**p3** (Fig. 1e): High-crowned tooth displays an irregular triangular shape, an occlusal outline with smooth anterior, internal and posterior borders covered by a thick enamel layer. External reentrant is deep, crossing about half of the total width of the tooth, crenulated as in the nominative taxon, and filled with deep cement. The anteroconid of quadrangular shape is much narrower than the posteroconid. The latter is wide, with rounded internal and posterior margins.

**p4** (Fig. 1f): High-crowned, rooted tooth; the trigonid is wider than talonid, they are connected by dense cement. The enamel band is well developed across all margins of the trigonid except for the anterior one; in the talonid the enamel layer is thin only on the small part of the antero-external corner.

### Discussion

*Amphilagus plicadentis* from Tagay is similar to the nominative form discovered in Unkheltseg/Mongolia (fauna UNCH-A/3/M; Early Miocene, biozone D) of the Valley of Lakes in Central Mongolia. In the upper teeth, the partial hypsodony is characteristic for all taxa of the amphilagine group. *A. plicadentis* from Tagay and UNCH-A/3 are similar in size and morphology of the p3 in having a deep external reentrant with plication. However, the Mongolian P3 is slightly larger than the P3 from Tagay, probably due to different individual age.

The Tagay *A. plicadentis* is locally close to *Amphilagus tomidai* found as well in the another Miocene Aya Cave site in the Baikal region.

*A. plicadentis* is only slightly smaller than *A. tomidai* but they differ much in tooth morphology: in *A. plicadentis* the anteroloph of the internal cone of P3 is long, distinguished to the mid-tooth width in contrast to that of *A. tomidai*, which shows a shorter anteroloph. The middle cone is large with an anterior triangular bulbous shape and a narrow isthmus posteriorly. The external cone of P3 is smaller than that in *A. tomidai,* and it is separated from the middle cone by a reentrant without cement. In contrast, in *A. tomidai* this external reentrant as well as the internal reentrant is filled with dense cement. P4 and M1 of *A. plicadentis* show on the occlusal surface an enamel islet, probably a rudiment of earlier superficial hypostria, which diminished with tooth wear. In contrast, in P4-M2 of *A. tomidai* no enamel islet is found on the occlusal surface. However, they do not have deep and short hypostria filled with thick cement. The parafossetes of these teeth are well developed and covered by deep cement. *A. plicadentis* differs from *A. tomidai* by having plication in the antero-external reentrant of p3 as well as by a slightly elongated and narrower anteroconid. Moreover, the talonid in p4-m2 is smaller than that in *A. tomidai*, and they all have a concave occlusal surface in contrast to those of the latter species. It is possible that *A. tomidai* could represents a slightly younger form than *A. plicadentis.* The Aya Cave site is located on the Olkhon plateau of the western Baikal coast, south-west from the Tagay locality (Daxner-Höck et al. 2022d). In the past, these two sites, Tagay and Aya, probably belonged to one united area. The transect of Aya Cave in Erbajeva and Filippov (1997, fig. 2; Filippov et al., 1995) evidences sediments of different ages. The fossil bearing sequence (layers 1-7) are composed of basal shallow lake sediments similar with sediments from the Tagay sequence (layers 1-2; Early/Middle Miocene transition). The large Cricetodontinae, Dipodidae, “*Rana*”, “*Trionyx*” and Lagomorpha were collected from the middle part (layers 3-5; Middle Miocene) and layers 6-7 yielded fragmentary bones of “Microtidae” (Plio–Pleistocene). The presence of the Mongolian taxon in the Siberian fauna suggests that the Baikalian region and Mongolia was one bioprovince, sharing a similar fauna and probability similar palaeoenvironment during the Early/Middle Miocene. *A. plicadentis* from Tagay differs from the other Eurasian *Amphilagus* species by its relatively smaller size (Tab. 2), having a plicated p3, a quadrangle anteroconid and a very wide and sharp posteroconid. *Amphilagus* appeared in Europe at the end of the late Oligocene with *Amphilagus wuttkei* Mӧrs and Kalthoff, 2010 representing the earliest record of this genus found in Enspel, Germany (Mӧrs and Kalthoff 2010) and *Amphilagus antiquus* Pomel, 1853 in the slightly younger Coderet locality in France (Tobien 1974) and in the recently discovered Herrlingen 9 locality in Baden-Württemberg, Germany (Fostowicz-Frelik 2016). The authors assume that favourable conditions at the end of Oligocene - beginning of Miocene allowed the genus *Amphilagus* to diversify and disperse widely. At that time, it dispersed from Western Europe into Eastern Europe, to Ukraine and Moldavia (Topachevsky 1987), and later on proceeded to Asia: Junggar Basin, North Western China and Japan in the Far East (Wu et al. 1998, Tomida and Goda 1993). *Amphilagus* extended to the Zaisan basin of Kazakhstan (Erbajeva 1994), the Baikal region of Siberia (Erbajeva and Filippov 1997, Erbajeva et al. 2016) and Mongolia (Erbajeva 2013).

The analysis of European and Asian taxa from Unkheltseg/Mongolia and of *A. tomidai* from the Middle Miocene of Siberia allows us to trace some changes in the evolutionary development. The main trend was enlargement of the dentition, increasing hypsodonty and an increasing amount of cement on the upper teeth (Erbajeva et al. 2016). The lower dentition (p4-m2) remained essentially the same, although a trend to increase the talonid width is seen.

## Supplementary Information

ESM 1 (DOCX 15.9 kb)

## Data Availability

All data generated or analysed during this study are included in this published article [and its supplementary information files].
