# Supplementary material for: Amphilagus plicadentis (Lagomorpha, Mammalia) from the Tagay locality (Olkhon Island, Baikal region, Eastern Siberia)
Source: Paleobiodivers Paleoenviron. 2022 Nov 28;102(4):915–20. doi: 10.1007/s12549-022-00554-y (PMC9758082; doi:10.1007/s12549-022-00554-y)
Supplement: Supplementary file 1 — (DOCX 15.9 kb) [file 12549_2022_554_MOESM1_ESM.docx]

Appendix 1. List of examined specimens

| Nr. | Faunistic horizon | Specimens  (all isolated) | Inventary numbers | Remarks |
| --- | --- | --- | --- | --- |
| 1 | Horizon 9 | R P3 | GIN Nr 2014/0003/1 |  |
| 2 | Horizon 9 | R P4 | GIN Nr 2014/0003/2 | Damaged |
| 3 | Horizon 9 | R P4 | GIN Nr 2014/0003/3 | Senile |
| 4 | Horizon 9 | L P4 | GIN Nr 2014/0003/4 | Juvenile |
| 5 | Horizon 10 | R P4 | GIN Nr 2014/0003/5 |  |
| 6 | Horizon 7 | R P4 | GIN Nr 2014/0003/6 |  |
| 7 | Horizon 7 | R M1 | GIN Nr 2014/0003/7 |  |
| 8 | Horizon 9 | R M2 | GIN Nr 2014/0003/8 |  |
| 9 | Horizon 9 | L p3 | GIN Nr 2014/0003/9 |  |
| 10 | Horizon 9 | R p4 | GIN Nr 2014/0003/10 |  |
| 11 | Horizon 7 | L p4 | GIN Nr 2014/0003/11 |  |
| 12 | Horizon 9 | R m1 | GIN Nr 2014/0003/12 |  |
| 13 | Horizon 9 | R m1 | GIN Nr 2014/0003/13 |  |
| 14 | Horizon 9 | L m1 | GIN Nr 2014/0003/14 |  |
| 15 | Horizon 9 | R m2 | GIN Nr 2014/0003/15 |  |
| 16 | Horizon 9 | L trigonid m1 | GIN Nr 2014/0003/16 |  |
| 17 | Horizon 9 | R I^1^ | GIN Nr 2014/0003/17 | Damaged |
| 18 | Horizon 9 | R I^2^ | GIN Nr 2014/0003/18 |  |
| 19 | Horizon 10 | R I_1_ | GIN Nr 2014/0003/19 |  |
| 20 | Horizon 10 | L I_1_ | GIN Nr 2014/0003/20 |  |
